# Robust growth of two-dimensional metal dichalcogenides and their alloys by active chalcogen monomer supply

**DOI:** 10.1038/s41467-022-28628-7

**Published:** 2022-02-23

**Authors:** Yonggang Zuo, Can Liu, Liping Ding, Ruixi Qiao, Jinpeng Tian, Chang Liu, Qinghe Wang, Guodong Xue, Yilong You, Quanlin Guo, Jinhuan Wang, Ying Fu, Kehai Liu, Xu Zhou, Hao Hong, Muhong Wu, Xiaobo Lu, Rong Yang, Guangyu Zhang, Dapeng Yu, Enge Wang, Xuedong Bai, Feng Ding, Kaihui Liu

**Affiliations:** 1 State Key Laboratory for Mesoscopic Physics, Frontiers Science Centre for Nano-optoelectronics, School of Physics, Peking University, 100871 Beijing, China; 2 Beijing National Laboratory for Condensed Matter Physics, Institute of Physics, Chinese Academy of Sciences, 100190 Beijing, China; 3 The Key Laboratory of Unconventional Metallurgy, Ministry of Education, Faculty of Metallurgical and Energy Engineering, Kunming University of Science and Technology, Kunming, 650093 Yunnan China; 4 Department of Physics and Beijing Key Laboratory of Opto-electronic Functional Materials&Micro-nano Devices, Renmin University of China, 100872 Beijing, China; 5 Centre for Multidimensional Carbon Materials, Institute for Basic Science, Ulsan, 44919 South Korea; 6 International Centre for Quantum Materials, Collaborative Innovation Center of Quantum Matter, Peking University, 100871 Beijing, China; 7 Songshan Lake Materials Laboratory, Dongguan, 523808 Guangdong China; 8 School of Physics and Telecommunication Engineering, South China Normal University, Guangzhou, 510006 Guangdong, China; 9 Interdisciplinary Institute of Light-Element Quantum Materials and Research Centre for Light-Element Advanced Materials, Peking University, 100871 Beijing, China; 10 Shenzhen Institute for Quantum Science and Engineering, Southern University of Science and Technology, Shenzhen, 518055 Guangdong China; 11 School of Physics, Liaoning University, Liaoning, 110136 Shenyang China

**Keywords:** Two-dimensional materials, Two-dimensional materials

## Abstract

The precise precursor supply is a precondition for controllable growth of two-dimensional (2D) transition metal dichalcogenides (TMDs). Although great efforts have been devoted to modulating the transition metal supply, few effective methods of chalcogen feeding control were developed. Here we report a strategy of using active chalcogen monomer supply to grow high-quality TMDs in a robust and controllable manner, e.g., MoS_2_ monolayers perform representative photoluminescent circular helicity of ~92% and electronic mobility of ~42 cm^2^V^−1^s^−1^. Meanwhile, a uniform quaternary TMD alloy with three different anions, i.e., MoS_2(1-*x*-*y*)_Se_2*x*_Te_2*y*_, was accomplished. Our mechanism study revealed that the active chalcogen monomers can bind and diffuse freely on a TMD surface, which enables the effective nucleation, reaction, vacancy healing and alloy formation during the growth. Our work offers a degree of freedom for the controllable synthesis of 2D compounds and their alloys, benefiting the development of high-end devices with desired 2D materials.

## Abstract (web summary)

The large-area growth of 2D transition metal dichalcogenides (TMDs) requires a precise control of metal and chalcogen precursors. Here, the authors implement a strategy using active chalcogen monomer supply to grow monolayer TMDs and their alloys, showing low defect density and improved optoelectronic properties.

## Introduction

Two-dimensional (2D) transition metal dichalcogenides (TMDs), with their atomic thicknesses, high carrier mobility, fast charge transfer, and intrinsic spin-valley couplings, have been demonstrated one of the most appealing candidates for next-generation electronic and optoelectronic devices^1–3^. The wafer-scale synthesis of TMDs with well-controlled crystallinity, quality, and composition is essential to fully realize their promising applications^4–13^. However, it is well known that the controllable growth of multi-element bulk materials is generally much more challenging than that of single-element ones. For example, the synthesized first-generation semiconductor, silicon, can have the extremely low impurity of ~10^−11^ and is nearly threading dislocation free, but the synthesized third-generation semiconductor, GaN, generally has a much higher impurity, ~10^−4^, and a threading dislocation density of ~10^4^–10^5^ cm^−2^ (ref. ^14^). Analogously for the growth of 2D materials, the as-grown graphene already has excellent properties, which is comparable to the samples exfoliated from natural crystals, and the measured carrier mobilities are close to the theoretical limit^15^, while the as-grown 2D compounds of TMDs, typically have lower quality than the natural crystals or the theoretical expectations^16^. The main difficulty in controllable compounds’ growth lies in the complicated feeding of several elements simultaneously during the growth process. Therefore, in the semiconductor industry, advanced and expensive techniques such as molecular beam epitaxy (MBE) and metal-organic chemical vapour deposition (MOCVD) have been developed to realize the precise control of multi-element supplies for the compound film growth.

Similarly, the synthesis of high-quality TMD materials requires the precise feeding control of both the transition metal and chalcogen precursors. In the past decade, intensive efforts have been devoted to optimizing the feeding of metal precursors by thermal evapouration or molten-salt-assisted evapouration of metal oxide^17^, decomposition of metal-organic precursor^18^, direct deposition of metal layers, and others^19–21^. Although some methods for controllable chalcogen feeding, such as using either elemental chalcogen or chalcogen compounds (i.e., heating sulfur powder, using H_2_S gas and ammonium sulfide^22–24^), have also been developed, it turns out that the chalcogen feeding control is much less effective than metal feeding control, as indicated by the most challenging problem in TMD quality control that the most synthesized TMDs are rich with chalcogen vacancies^25^. Therefore, developing more effective chalcogen supply methods to enable the growth of TMDs with high quality and rich composition is of critical importance.

In this work, we propose to use a chalcogen monomer feeding method in the controllable TMD growth because of the following potential advantages. (i) The chalcogen monomers or atoms are generally very active than the corresponding dimers or bulks and thus they can quickly react with metal precursors to form TMDs, (ii) the active chalcogen monomers can bind and quickly diffuse on a TMD surface to scavenge the vacancy defects effectively, which will greatly improve the quality of the TMDs and (iii) an active chalcogen monomer can react with a TMD and easily substitute a chalcogen atom and, thus, allow the synthesis of uniform TMD chalcogen alloys. However, as the monomer state of chalcogen only exists at very high temperature (>2500 K) under normal circumstance^26^, the most used methods can not produce enough chalcogen monomers at the typical TMD growth temperature, which is generally <~1300 K. Herein, we developed an effective route to provide chalcogen monomer by heating a metal chalcogenide. The success of this approach lies in that the chalcogen atoms on the surface of metal chalcogenides can be easily released in the chalcogen atom (monomer) form under a relatively lower temperature^27,28^. The slowly released chalcogen monomers have a very low probability to react with each other to form dimers, thus, enable the successful synthesis of TMDs (MX_2_, M = Mo, W; X = S, Se, Te) and their alloys with very high quality.

## Results

### MoS_2_ growth by sulfur monomer supply

**Fig. 1:**
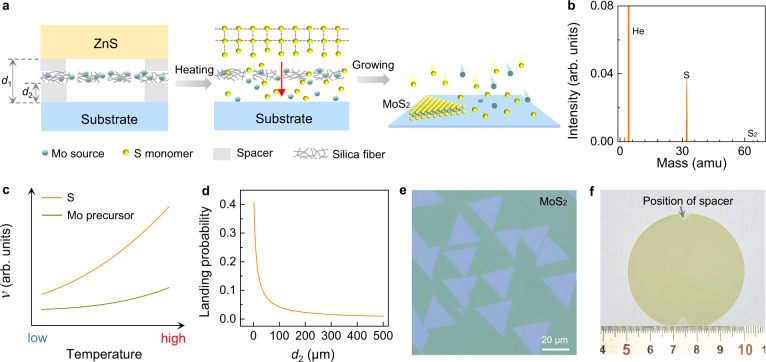
Growth of wafer-scale monolayer MoS_2_ by sulfur monomer supply. **a** Schematic of sulfur (S) monomer supply for the growth of MoS_2_. At high temperature, the released S monomers from the surface of ZnS and the vaporized Mo source from the precoated silica fiber fabric can penetrate the porous fabric and form monolayer MoS_2_ on the target substrate. *d*_1_ and *d*_2_ are the distances from the substrate to the ZnS and the silica fiber fabric, respectively. The red arrow denotes the diffusion of S monomers released from the ZnS surface. **b** In-situ mass spectrum of ZnS annealed at 1000 °C. The intense peak at the mass of 32 clearly proves the dominating release of S monomers. The measurements were carried out with carrier gas of He and the data was subtracted by background. **c** The illustration of the temperature-dependent release rate of S monomers (orange curve) and evapouration rate of Mo precursor (dark yellow curve). **d** The landing probability of Mo precursor (orange curve) as a function of *d*_2_ as modelled in Supplementary Note 2. **e** Optical image of the as-grown monolayer MoS_2_ domains on sapphire when *d*_2_ is 100 μm. **f** Photograph of a 2-inch monolayer MoS_2_ film on sapphire when *d*_2_ is 20 μm. The uncovered regions are the positions of the mica spacers.

In our design, the metal chalcogenide plate of ZnS, Na_2_MoO_4_ coated silica fibre fabric and the target substrate were vertically stacked as a sandwich structure by using mica spacers (Fig. 1a, left panel). The distance of *d*_1_ (*d*_2_) between the S (Mo) precursor and the growth substrate can be modulated by varying the thickness of mica (from tens to hundreds of microns), which is essential to tune the fluxes of S and Mo independently. At an elevated growth temperature (~750–950 °C), S monomers were released from the ZnS surface and Na_2_MoO_4_ started to vapourize (Fig. 1a, middle panel). Then both S and Mo sources passed through the porous fibre fabric (Supplementary Fig. 1) and reached to the substrate surface to form monolayer MoS_2_ (Fig. 1a, right panel). It is important to note that the S monomers can’t be obtained by the sublimation of sulfur powders, where S_2_ dimers are always the dominating species at thermal equilibrium^29^. Indeed, the release of S monomers from the metal sulfide surface was observed long time ago^30^, and was unambiguously revealed by the in-situ mass spectroscopy as shown in Fig. 1b.

In our experiment, we found that the flux of S monomers is significantly larger than that of the Na_2_MoO_4_ or the S is overfed (Fig. 1c and Supplementary Note 1), as we observed that the variation of *d*_1_ (the distance between the ZnS and the substrate) has limited effect on the MoS_2_ growth, while the variation of *d*_2_ (the distance between the fibre fabric and the substrate) affects the nucleation density of MoS_2_ domains greatly (Supplementary Fig. 2). This phenomenon can be qualitatively understood as that, by decreasing the *d*_2_, the landing probability of evaporated Mo source onto the substrate becomes larger (Fig. 1d and Supplementary Note 2), which can further boost the nucleation of MoS_2_ domains. As shown in Fig. 1e, f, sparse triangular domains and 2-inch continuous film of MoS_2_ can be respectively obtained after the same growth duration of 50 min when *d*_2_ was 100 μm and 20 μm. In the limit case, the *d*_2_ can also be zero by directly precoating a thin layer of Na_2_MoO_4_ precursor into the substrate (Supplementary Fig. 3).

### Quality characterizations of MoS_2_ domains

**Fig. 2:**
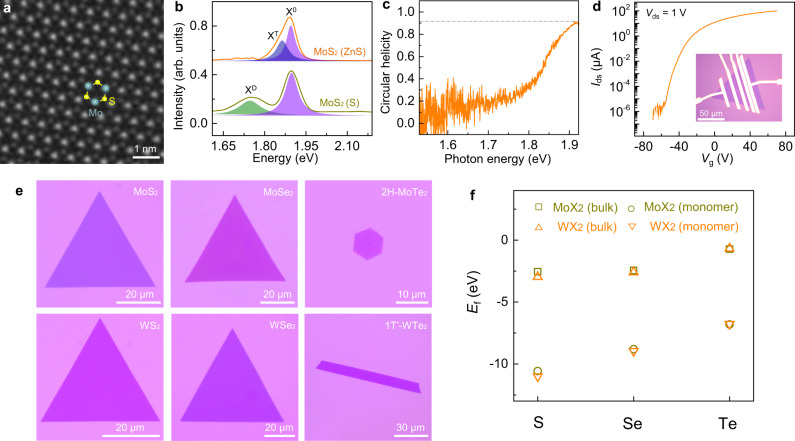
Quality characterizations of the as-grown MoS_2_ domain and universal growth of diverse TMDs by chalcogen monomer supply. **a** Atomic-resolved HAADF-STEM image of the prepared MoS_2_, revealing the high crystallinity of MoS_2_ without detectable S vacancies. **b** Low-temperature (10 K) PL spectra of MoS_2_ samples fed by S monomer (orange curve) and S powder (dark yellow curve), respectively. Three typical features, X^0^, X^T^, and X^D^ peaks assign to neutral exciton, trion, and defect state emission peaks, respectively. The absence of X^D^ peak confirmed the high quality of the MoS_2_ grown by S monomer supply. **c** The circular dichroism PL spectrum measured at 10 K. The near-unity polarization of MoS_2_ on sapphire indicates the high optical quality. The horizontal dashed line is added for clarity. **d** Transfer characteristic of the MoS_2_ FET with channel length and width of 7 μm and 22 μm, respectively, at a bias voltage *V*_ds_ of 1 V. Inset: optical image of the device. **e** Optical images of the representative TMDs, including 2H phase MoS_2_, MoSe_2_, MoTe_2_, WS_2_, WSe_2_ and 1T′ phase WTe_2_. **f** The calculated formation energy (*E*_f_) of the six representative TMDs. When chalcogen bulks are supplied as precursors, the formation of transition metal tellurides in relative to their corresponding sulfide and selenide are less favourable due to their high formation energies (−0.73 and −0.68 eV/unit for MoTe_2_ and WTe_2_, respectively). While it becomes highly favourable when Te monomers are applied.

A series of characterizations unequivocally reveal the high quality of MoS_2_ fed by S monomer supply. Atomic-resolved atomic force microscopic (AFM) images at different positions of the as-grown MoS_2_ clearly resolved the S atoms without obvious S vacancies (Supplementary Fig. 4). High-angle annular dark-field scanning transmission electron microscopic (HAADF-STEM) images exhibited the perfect hexagonal honeycomb lattice with both Mo and S atoms (Fig. 2a and Supplementary Fig. 5). And the density of S vacancy defects was extracted as ~2 × 10^12^ cm^−2^, which is among the lowest value in previous STEM measurements (Supplementary Table 1).

The low-temperature photoluminescence (PL) spectra of the S-monomer-feeding-grown MoS_2_ (Fig. 2b, orange curve) has a characteristic neutral exciton (X^0^) emission peak accompanied with a trion (X^T^) peak (believed to be caused by the n-type doping from substrate^31^), but the X^D^ peak (believed to be caused by S vacancy^32^ and was obvious in S-powder-feeding-grown samples, Fig. 2b, dark yellow curve) is nearly invisible, which clearly proves the high quality of the S-monomer-feeding-grown MoS_2_ samples. Meanwhile, the uniform distribution of the PL peak intensity and the narrow full width at half maximum both demonstrated the high uniformity and crystallinity of the sample grown by S monomer supply (Supplementary Fig. 6).

In addition, the high quality of as-grown MoS_2_ can be further confirmed by the measured optical circular helicity^33^, which was detected to be as high as 92% (Fig. 2c) and comparable with the best-exfoliated flakes from high-quality natural crystals. The circular helicity is directly related to the scattering between K and K′ valleys in the Brillouin zone of MoS_2_ whilst the defects will greatly enhance the inter-valley scattering and decrease the circular helicity value. Thus, the near-unity circular helicity strongly proves the high quality of the as-grown MoS_2_ samples.

We further verified the quality of the single-crystal MoS_2_ domain by evaluating its field-effect mobility in a bottom-gate transistor configuration. The output and transfer characteristics of a typical field-effect transistor (FET) devices with channel length/width (L/W) of 7/22 μm are exhibited in Fig. 2d and Supplementary Fig. 7. The device exhibits a typical n-type transfer characteristic with an on/off ratio of ~10^8^ at room temperature and electron field-effect mobilities of ~42 cm^2^V^−1^s^−1^, which are comparable to the performance of monolayer MoS_2_ prepared by mechanical exfoliation^34^. Statistics of the transport measurement are also given in Supplementary Fig. 8, demonstrating the good uniformity of the MoS_2_ electronic devices. These results suggest that S-monomer-feeding-grown MoS_2_ samples have appreciably high electronic quality.

### Universal TMD growth by chalcogen monomer supply

Our strategy on MoS_2_ growth by monomer feeding has also been proved to be applicable for the growth of various high-quality TMD materials. Six typical monolayer TMDs (Fig. 2e) have been successfully synthesized by simply replacing the transition metal sources (e.g., Na_2_MoO_4_ and Na_2_WO_4_) and chalcogenide plates (e.g., ZnS, ZnSe, and ZnTe) (see Methods and Supplementary Fig. 9). The Raman and PL spectra of these obtained TMD samples demonstrated the successful synthesis of the 2H phase MoS_2_, WS_2_, MoSe_2_, WSe_2_, MoTe_2,_ and the 1T’ phase WTe_2_ and MoTe_2_ (Supplementary Fig. 10). It is worth noting that, due to their higher formation energies in relative to the corresponding sulfide and selenide bulks (Fig. 2f and Supplementary Note 3), the growth of transition metal tellurides, e.g., MoTe_2_ and WTe_2_, by using chalcogen bulks as feedstocks is usually very challenging^35^. Thanks to the introduction of active Te monomers, the synthesis of WTe_2_ and MoTe_2_ is very easy and efficient because of the greatly reduced formation energies (Fig. 2f).

### Controllable synthesis of quaternary TMD alloy

**Fig. 3:**
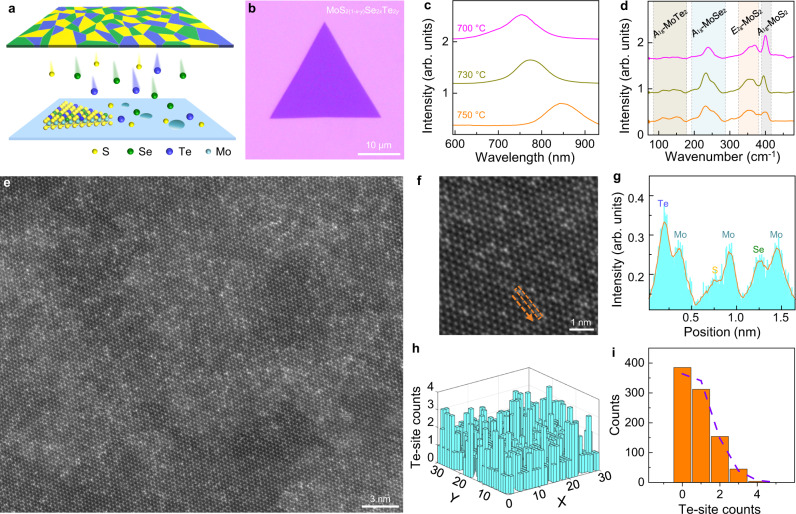
Growth and characterizations of quaternary TMD alloy. **a** Schematic diagram of quaternary alloy growth using a compressed plate mixed with chalcogenide powders of ZnS, ZnSe, and ZnTe. **b** Optical image of MoS_2(1-*x*-*y*)_Se_2*x*_Te_2*y*_ domain on SiO_2_/Si substrate. **c**, **d** PL (**c**) and Raman (**d**) spectra of the MoS_2(1-*x*-*y*)_Se_2*x*_Te_2*y*_ sample grown at different temperatures. As the growth temperature increased, the PL peak position showed a clear red shift. The intensity of MoS_2_-like *E*_2g_ (~380 cm^−1^) and *A*_1g_ (~400 cm^−1^) was reduced while the MoTe_2_-like *A*_1g_ (~150 cm^−1^) increased and MoSe_2_-like *A*_1g_ (~240 cm^−1^) increased first and then reduced. The shaded areas are added for clarity. **e**, **f** STEM images of the MoS_2(1-*x*-*y*)_Se_2*x*_Te_2*y*_, demonstrating the high crystallinity of quaternary alloy. **g** Intensity profile along the labeled orange dotted box in (**f**), which highlights the occupancies of Mo, S, Se, and Te sites. **h** The Te-site distribution in a 32 × 32 nm^2^ STEM image of the quaternary alloy. The image was divided into 30 × 30 parts. **i** The corresponding statistical histogram of Te-site counts in each parts of the image. It shows a well binomial distribution feature (purple dotted line), revealing the random distribution of Te atoms.

The chalcogen monomer feeding method has a unique advantage in the growth of TMD chalcogen alloys. Since the evapouration temperatures, saturated vapour pressures and reaction energies of S, Se, and Te are significantly different, it is nearly impossible to form high-quality TMD alloys with more than two anion elements by traditional CVD approaches^36^. Till now, there is no report on the successful growth of MoS_2(1-*x*-*y*)_Se_2*x*_Te_2*y*_ alloy. In our experiment, we applied a compressed plate mixed with different metal chalcogenide powders, i.e., ZnS, ZnSe, and ZnTe, to supply three kinds of chalcogen monomers (S, Se, and Te) simultaneously (Fig. 3a). The as-grown alloy of MoS_2(1-*x*-*y*)_Se_2*x*_Te_2*y*_ has a triangular domain similar to 2H phase TMDs (Fig. 3b). The X-ray photoelectron spectroscopy (XPS) unambiguously revealed the coexistence of S, Se, and Te atoms in the synthesized TMD alloy (Supplementary Fig. 11). The energy-dispersive X-ray spectroscopy (EDS) as well as the STEM measurements further demonstrated the homogenous element distribution throughout the TMD alloy in both macro- and micro-scales, with no observable phase separation (Fig. 3e and Supplementary Fig. 12). Enlarged STEM image (Fig. 3f) further demonstrated the high crystallinity of the TMD alloy. The distinct intensity distribution revealed the occupancies of Mo, S, Se, and Te according to the Z-contrast nature of HAADF image (Fig. 3g). Quantitative analysis of the Te and Se distribution is presented in a 32 × 32 nm^2^ STEM image, and the statistical results match well with the binomial distribution model (Fig. 3h, i, Supplementary Fig. 13, and Supplementary Note 4), suggesting a random distribution of S, Se and Te atoms in the TMD alloy.

Furthermore, one can easily tune the composition of the TMD alloys by controlling the growth temperature to vary the fluxes of S, Se, and Te from the ZnS, ZnSe, and ZnTe composite. As the growth temperature increased, the PL peak shifted to longer wavelength (Fig. 3c), revealing the increase of the concentrations of Se and Te in the alloy (MoSe_2_ and MoTe_2_ have smaller bandgaps than MoS_2_). Meanwhile, in the Raman spectra, the MoS_2_-featured peaks gradually attenuated, the MoTe_2_-featured peaks gradually enhanced, while the MoSe_2_-featured peaks enhanced first and then attenuated (Fig. 3d), which indicates that heavier chalcogen atoms are being doped into the alloy at a higher temperature.

### Mechanism for the chalcogen monomer-modulated TMD growth

Finally, we try to understand the unique role of chalcogen monomer supply in the synthesis of high-quality TMDs and their complex alloys theoretically. We firstly explore the reactions of Na_2_MoO_4_ with sulfur monomers and dimers, respectively, by first-principles molecular dynamic (MD) simulations (Supplementary Fig. 14). The simulation results clearly demonstrated that sulfur monomers are more reactive to substitute the oxygen atoms in a MoO_4_^2−^ group of Na_2_MoO_4_ while most dimers are desorbed from the Na_2_MoO_4_ surface due to their less activity (Supplementary Note 5). By adding more S monomers and MoS_3_ clusters to the Na_2_MoO_4_, the nucleation of Mo_*x*_S_*y*_ clusters can also be clearly seen (Supplementary Fig. 15).

**Fig. 4:**
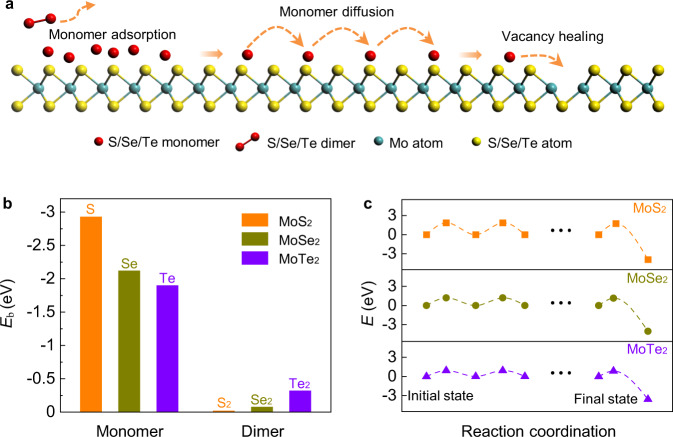
Growth mechanism with chalcogen monomer supply in MoX_2_ (X = S, Se, Te). **a** Schematic diagram of adsorption, diffusion, and vacancy healing of chalcogen monomer on MoX_2_ surface. The orange dotted arrows denote the motion of chalcogen monomers or dimers. **b** The binding energies of monomers and dimers on MoX_2_ surface. The much higher energy of monomers facilitates their better adsorption on the TMD surface than dimers. **c** The energy profiles of vacancy healing for MoX_2_ surface by using chalcogen monomers. The relatively small energy barriers of chalcogen monomer diffusion and the highly exothermic reaction at the vacancy both accelerate the self-healing of MoX_2_.

In compare with the bond-saturated S_2_/Se_2_/Te_2_ dimers, S/Se/Te monomers possess much higher adsorption energies on a TMD surface (for dimers *E*_b_ < 0.8 eV, for monomers *E*_b_ > 1.5 eV) (Fig. 4b and Supplementary Fig. 16a). Therefore, one can expect a large number of S/Se/Te monomers to diffuse on the TMD surface during the whole growth process and the growth of TMD is in a chalcogen monomer rich environment. Once a chalcogen monomer diffuses to the vicinal area of a vacancy, the vacancy can be quickly healed by a highly exothermic reaction (Fig. 4a, c and Supplementary Fig. 16b). Therefore, the chalcogen vacancy density is greatly reduced as has been shown in the experimental results. To address the capacity of forming TMD chalcogen alloys, we calculated the reaction energy of substituting a chalcogen atom in a TMD by using chalcogen monomer, dimer, and bulk as references (Supplementary Fig. 17 and Supplementary Note 6). It is clearly shown that doping of Te dimer or bulk into MoS_2_/MoSe_2_, S bulk into MoSe_2_, Se bulk into MoS_2_ are all difficult because of the near-zero or positive reaction energies. While, if chalcogen monomer is used as the source of dopant, all doping reactions become exothermic with noticeable negative reaction energies, which implies the greatly improved capacity of forming chalcogen TMD alloys.

## Discussion

This study clearly demonstrated that the high reactivity of chalcogen monomers can significantly facilitate the TMD nucleation, chalcogen defect healing in the growth to greatly improve the samples’ quality and allow the formation of various chalcogen TMDs and their alloys. The monomer supply should provide a degree of freedom in modulating compound materials and high-entropy 2D alloys, thus widening their potential applications in electronic, optoelectronic and valleytronic devices.

## Methods

### Growth of TMDs and their alloys

The substrate (sapphire or SiO_2_/Si) and silica fibre fabric were first pretreated with O_2_ plasma. Then the silica fibre fabric was immersed in Na_2_MoO_4_ or Na_2_WO_4_ aqueous solution with optimized concentrations (Na_2_MoO_4_ of 12, 18, and 6 mg/mL for MoS_2_, MoSe_2_, and MoTe_2_ growth, Na_2_WO_4_ of 20, 30, and 12 mg/mL for WS_2_, WSe_2_, and WTe_2_ growth, respectively). After dried in Ar atmosphere, silica fibre fabric and the chalcogenide crystal plate (ZnS, ZnSe, or ZnTe) were placed above the substrate in sequence by using two pieces of mica as the spacers respectively, and then loaded into the CVD furnace together. The chamber of furnace was flushed with Ar (100 sccm) and heated to the optimized growth temperature (~780 °C, ~930 °C, ~800 °C, ~820 °C, ~750–800 °C, ~800 °C, and ~780 °C for MoS_2_, WS_2_, MoSe_2_, WSe_2_, 2H-MoTe_2_, 1 T′-MoTe_2_, and 1 T′-WTe_2_, respectively). During the growth process, the system pressure was kept at ~120 Pa and the growth duration was set as 10-60 min. After growth, the system was naturally cooled down to room temperature. Similar growth conditions were applied to the TMD alloy growth, wherein the major difference lies in the use of a chalcogenide mixture plate.

### Sample characterizations

Optical images were taken with an Olympus BX51M microscope. Raman and PL spectra were measured by a customer-designed optical system with the excitation wavelength of 532 nm and the power of ~1 mW. Low-temperature PL spectra were obtained at 10 K by optical cryostat (Montana Instruments) with the laser excitation wavelength of 532 nm. Circular-polarization-resolved PL measurements were performed under near-resonant excitation of 633 nm at 10 K. The circularly polarized light was generated by using a super-achromatic quarter-wave plate (Thorlabs SAQWP05M-1700) and the photoluminescence was analyzed through the same quarter-wave plate and a linear-polarizer. We define the degree of PL circular helicity (*η*), which reflects the valley polarization, as *η* = [PL(*σ*^+^)−PL(*σ*^−^)]/[PL(*σ*^+^)+PL(*σ*^−^)]. XPS measurements were performed using an ESCALAB 250X system (Thermo Fisher Scientific) and excited by monochromatic Al Kα radiation. Mass spectrometer (Hiden HR20) attached with temperature-programmed decomposition (TPD) was used to in-situ detect and analyze the released gas in inert atmosphere. EDS and STEM experiments were performed in FEI Titan Themis G2 300 operated at 300 kV and in Nion U-HERMES200 at 60 kV for element analysis and characterizing atomic structures of samples. Atomic-resolved AFM measurements were performed using Asylum Research Cypher in ambient atmosphere.

### Fabrication and measurement of MoS_2_ FET device

Electron-beam lithography (EBL) is used to define the channel and the source/drain contacts with PMMA EBL resists. Metallization is implemented by thermal evapouration of 20 nm bismuth with a rate of 0.2 Å s^−1^, followed by an Au capping layer by electron-beam evapouration (30 nm at 0.1 Å s^−1^) at ~10^−7^ torr. Lift-off process is carried out in hot acetone. All electrical characterization is conducted in a vacuum environment and room temperature in a Janis probe station using a semiconductor device analyser (Agilent Technologies B1500A). The field mobilities of MoS_2_ were calculated according to the equation *μ* = [d*I*_ds_/d*V*_g_] × [*L*/(*WC*_i_*V*_ds_)]. In this equation, the *L* and *W* are the channel length and width, respectively. *C*_i_ is the capacitance per unit area of 300 nm SiO_2_ dielectric layer, 11.5 × 10^−5^ F.

### Computational details

All the density functional theory (DFT) calculations were implemented by the Vienna Ab Initio Simulation Package (VASP)^37,38^, with projector-augmented wave (PAW)^39^ method describing the interaction between valence electrons and ion cores. The Perdew-Burke-Ernzerhof (PBE)^40,41^ exchange-correlation functional was used to describe the interaction between electrons. A plane wave basis set with a cutoff energy of 450 eV was adopted. All the structures were fully relaxed, and the convergence criteria for energy and force were set at 10^−5^ eV and 10^−2^ eV/Å, respectively. The Brillion zone is sampled by 1 × 1 × 1 grid meshes. A vacuum spacing larger than 15 Å was set to avoid the interaction between neighbouring images along the non-periodic direction. The energy barriers were calculated by using the climbing image nudged elastic band (CI-NEB) method^42^ with a force threshold of −0.02 eV/Å.

## Supplementary information

Supplementary Information

## Data Availability

The authors declare that the data supporting the findings of this study are available within the paper, Supplementary Information and Source Data. Extra data are available from the corresponding authors upon request. Source data are provided with this paper.

## Source data

Source Data
