# Supplementary material for: Robust growth of two-dimensional metal dichalcogenides and their alloys by active chalcogen monomer supply
Source: Nat Commun. 2022 Feb 23;13:1007. doi: 10.1038/s41467-022-28628-7 (PMC8866400; doi:10.1038/s41467-022-28628-7)
Supplement: Supplementary file 1 — Supplementary Information [file 41467_2022_28628_MOESM1_ESM.pdf]

Supplementary Information for

# Robust growth of two-dimensional metal dichalcogenides and their alloys by active chalcogen monomer supply

Yonggang Zuo, Can Liu, Liping Ding, Ruixi Qiao, Jinpeng Tian, Chang Liu, Qinghe Wang, Guodong Xue, Yilong You, Quanlin Guo, Jinhuan Wang, Ying Fu, Kehai Liu, Xu Zhou, Hao Hong, Muhong Wu, Xiaobo Lu, Rong Yang, Guangyu Zhang, Dapeng Yu, Enge Wang, Xuedong Bai, Feng Ding, Kaihui Liu

**The supplementary information includes:**

**Supplementary Fig. 1-17**

**Supplementary Table 1**

**Supplementary Note 1-6**

**References**

# Table of Contents


**Supplementary Figures** 3

1: Optical image of the silica fibre fabric after the $MoS_2$ growth 3

2: Gap distance-dependence of the $MoS_2$ nucleation density on sapphire 4

3: Schematic of S monomer supply for the growth of $MoS_2$ when $d_2$ is zero 5

4: Atomic-resolved atomic force microscopic (AFM) images of the $MoS_2$ 6

5: Atomic-resolved scanning transmission electron microscopic (STEM) images of the $MoS_2$ 7

6: Photoluminescence (PL) of the S-monomer-feeding-grown $MoS_2$ 8

7: Electronic measurements of the as-grown monolayer $MoS_2$ domain 9

8: Statistical results of the $MoS_2$ electrical performance at room temperature 10

9: In-situ mass spectrum of ZnSe annealed at 1000 °C. 11

10: 1T'-$MoTe_2$ optical image and optical measurements of diverse TMDs 12

11: X-ray photoelectron spectroscopy (XPS) of quaternary $MoS_{2(1-x-y)}Se_{2x}Te_{2y}$ alloy 13

12: Energy Dispersive X-ray Spectroscopy (EDS) of quaternary $MoS_{2(1-x-y)}Se_{2x}Te_{2y}$ alloy 14

13: STEM analysis of quaternary 2D $MoS_{2(1-x-y)}Se_{2x}Te_{2y}$ alloy 15

14: Molecular dynamic (MD) simulation of sulfidation of liquid $Na_2MoO_4$ 16

15: MD simulation on the nucleation process with S monomers supply 17

16: Proposed mechanism of chalcogen monomer modulated $WX_2$ growth 18

17: Calculated reaction energies of trinary TMD alloys 19

**Supplementary Table** 20

1: Reported S vacancy density of as-grown monolayer TMDs 20

**Supplementary Notes** 21

1: Demonstrations on the supply rates of S monomer and Mo source 21

2: Theoretical calculations on the landing probability of Mo precursor onto the substrate 22

3: Calculations of the formation energies of different transition metal dichalcogenides 23

4: Statistical Se and Te atom distribution in the quaternary $MoS_{2(1-x-y)}Se_{2x}Te_{2y}$ alloy 24

5: Calculations on initiated nucleation of $MoS_2$ by S monomer or dimer supply 26

6: Reaction energies of ternary TMD alloys obtained by X (X = S, Se, Te) doped $MoX_2$ 27

**References** 28

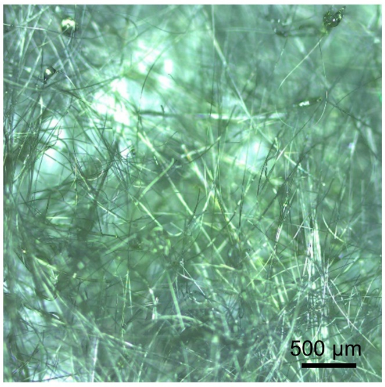


**Supplementary Fig. 1 | Optical image of the silica fibre fabric after the $MoS_2$ growth.**

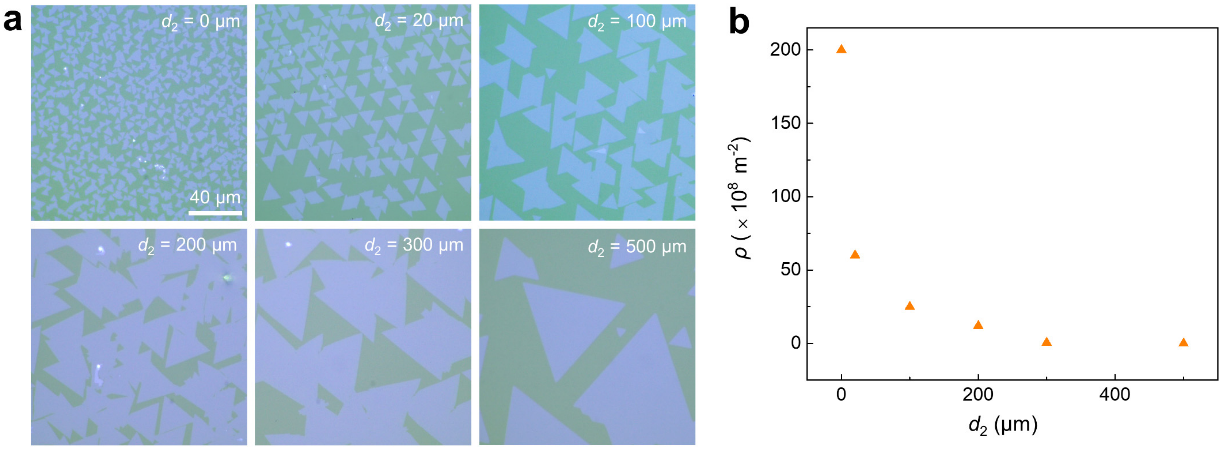


**Supplementary Fig. 2 | Gap distance-dependence of the $MoS_2$ nucleation density on sapphire.** **a**, Optical images of the as-grown $MoS_2$ when $d_2$ varied is from 0 to 500 μm. **b**, The nucleation density of the as-grown $MoS_2$ (orange triangle) as a function of the gap distance of $d_2$.

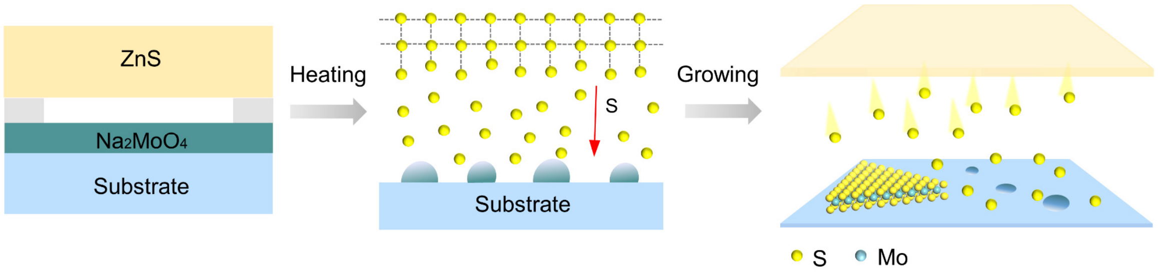


**Supplementary Fig. 3 | Schematic of S monomer supply for the growth of $MoS_2$ when the $Na_2MoO_4$ was directly spin-coated on the surface of the substrate, in which $d_2$ is zero.**

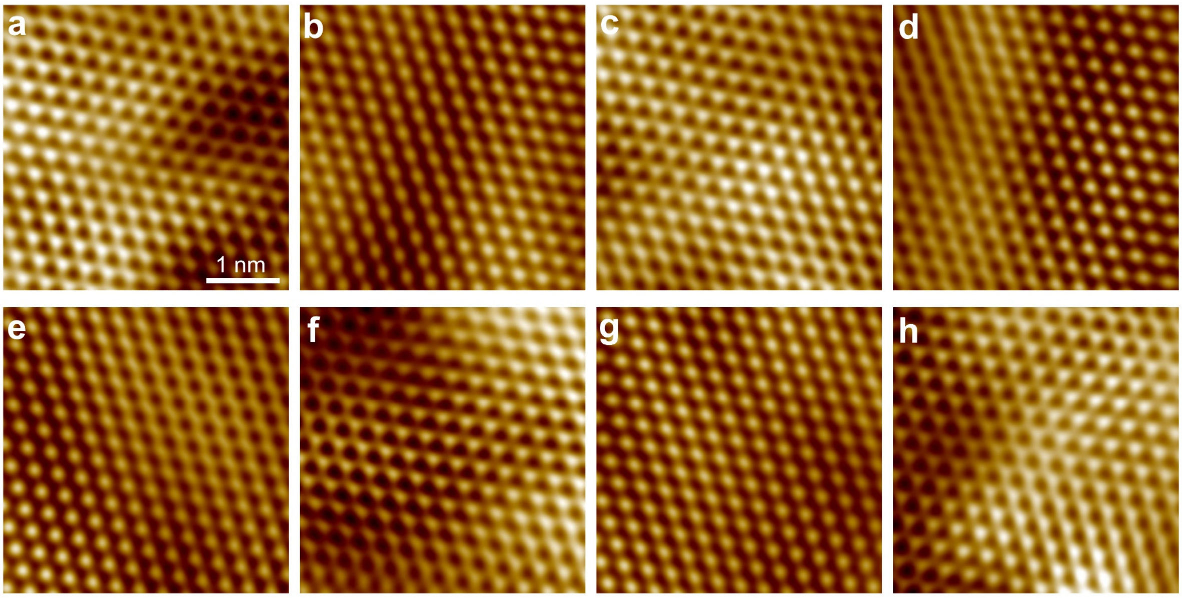


**Supplementary Fig. 4 | Atomic-resolved atomic force microscopic (AFM) images of the as-grown $MoS_2$ samples.** The S atoms are clearly resolved by AFM, which doesn't show any obvious S vacancies, confirming the ultra-low defect density in our samples.

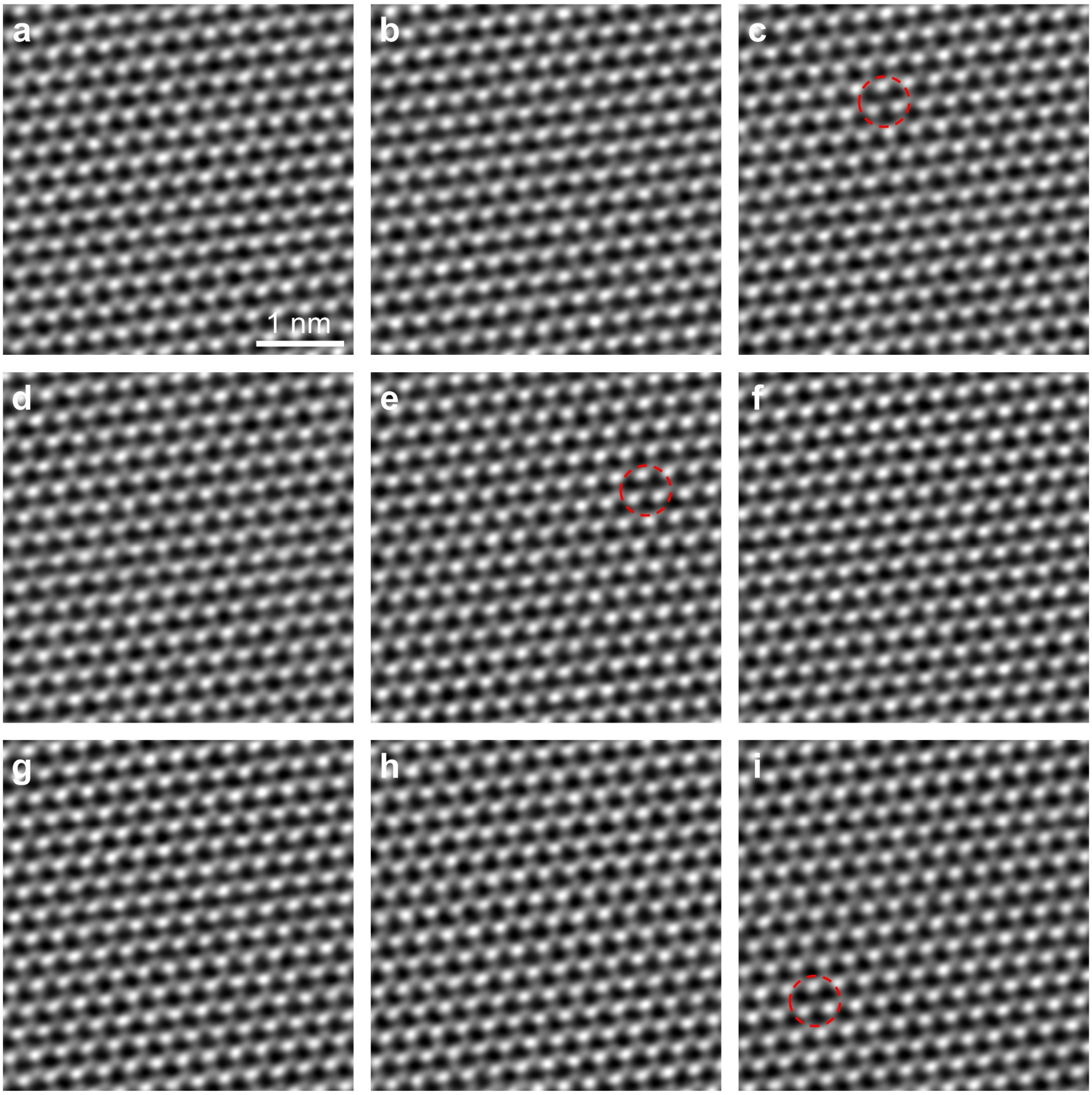


**Supplementary Fig. 5 | Atomic-resolved scanning transmission electron microscopic (STEM) images captured at different regions of as-grown monolayer $MoS_2$.** All of the images showed perfect hexagonal honeycomb lattice structure, while the low contrast sites marked by red dotted circles indicated the occasional S vacancy. The defect density was extracted at the order of $\sim 2\times 10^{12}$ $cm^{-2}$.

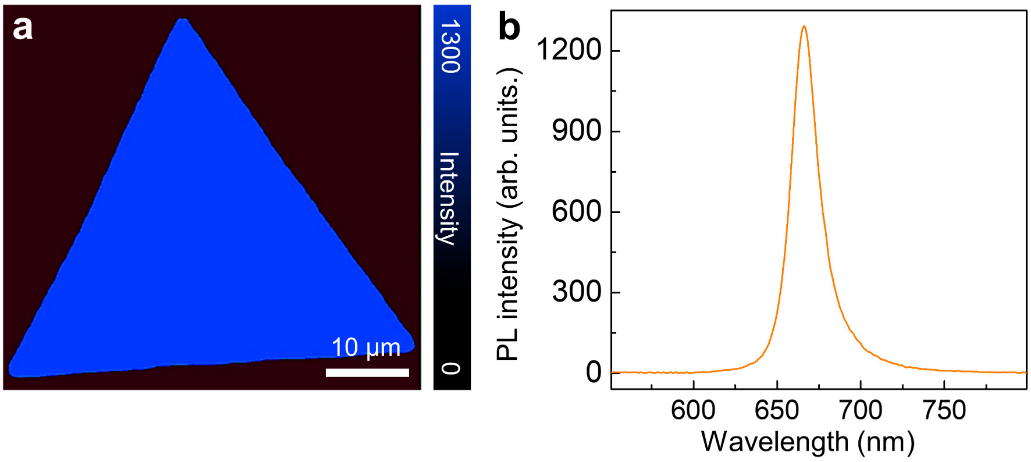


**Supplementary Fig. 6 | Photoluminescence (PL) of the S-monomer-feeding-grown $MoS_2$. a**, The PL mapping image of a whole single-crystal $MoS_2$ domain grown by S monomer supply. **b**, A representative PL spectrum of the as-grown $MoS_2$. The sharp PL peak at 667 nm (with a narrow full-width at half-maximum of 53 meV) demonstrated the high crystallinity of the sample grown by S monomer supply.

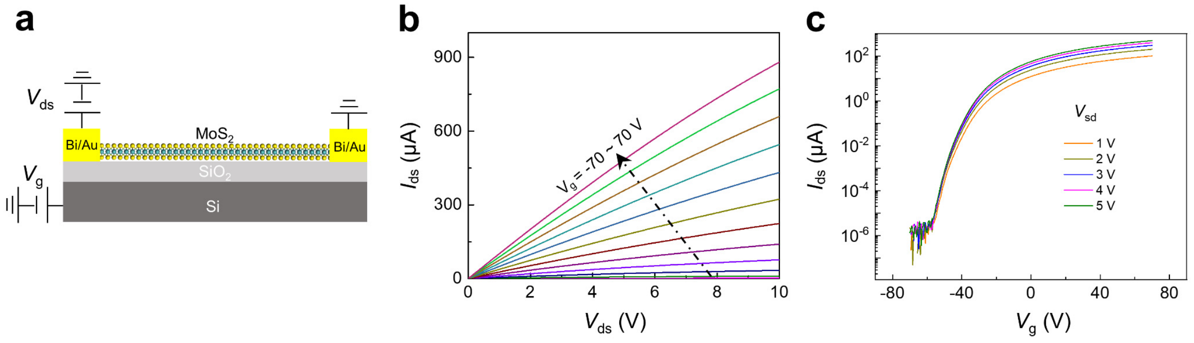


**Supplementary Fig. 7 | Electronic measurements of the as-grown monolayer $MoS_2$ domain on $SiO_2$/Si substrate. a**, Schematic of the monolayer $MoS_2$ FET device structure. **b**, Output characteristics of the FET device with gate voltage sweeping from -70 to 70 V. **c**, Transfer characteristics of the device with gate voltage sweeping from -70 to 70 V when the bias voltage is varied from 1 to 5 V. The on/off rate is ~$10^8$ and the threshold voltage is around -60 V.

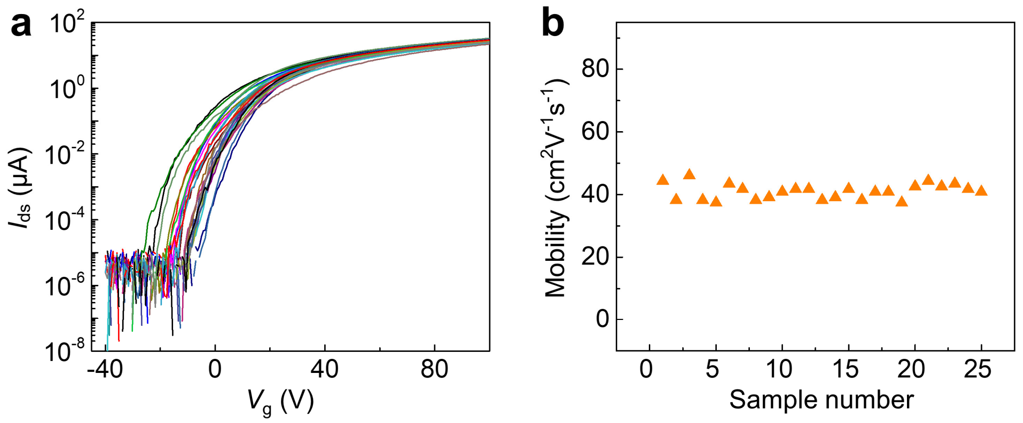


**Supplementary Fig. 8 | Statistical results of the $MoS_2$ electrical performance at room temperature. a**, Transfer curves of 25 randomly-selected $MoS_2$ FETs ($L/W = 1$) with the channel length and width of 5 μm and 5 μm respectively, at a bias voltage $V_{ds}$ of 1V. **b**, The corresponding statistics of $MoS_2$ FET mobility, showing the average mobility of ~41 $cm^2V^{-1}s^{-1}$.

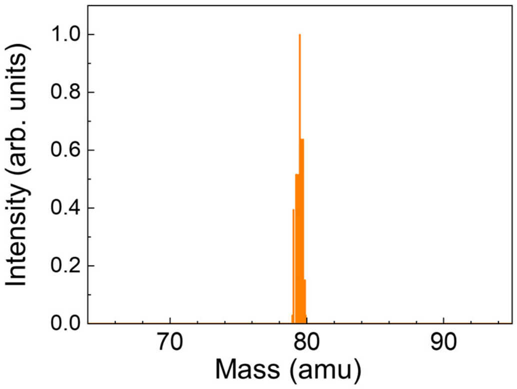


**Supplementary Fig. 9 | In-situ mass spectrum of ZnSe annealed at 1000 °C.**

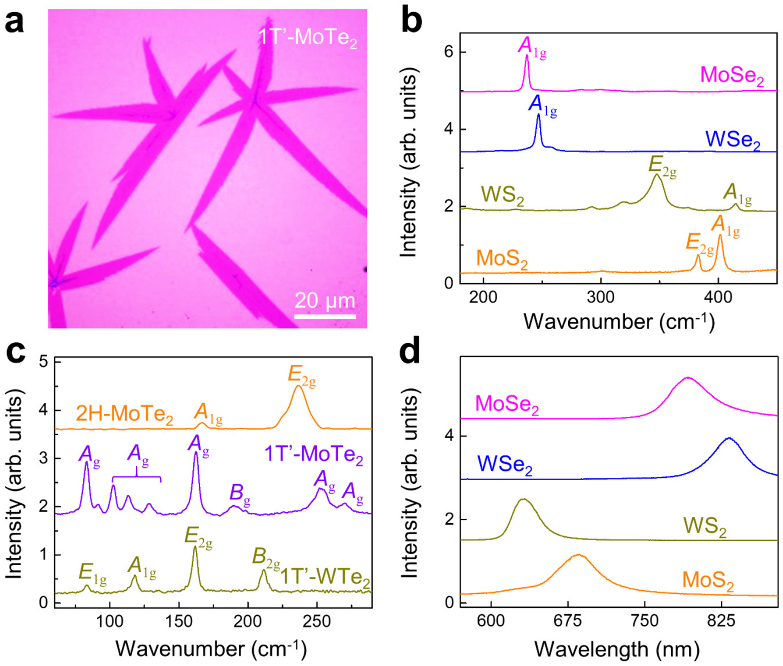


**Supplementary Fig. 10 | 1T'-MoTe$_2$ sample prepared by Te monomer supply and optical measurements of diverse TMDs prepared by chalcogen monomers supply. a**, Optical image of 1T'-MoTe$_2$. **b-d**, The corresponding Raman (b,c) and PL (d) spectra of as-grown TMDs. All spectra were vertically shifted for clarity.

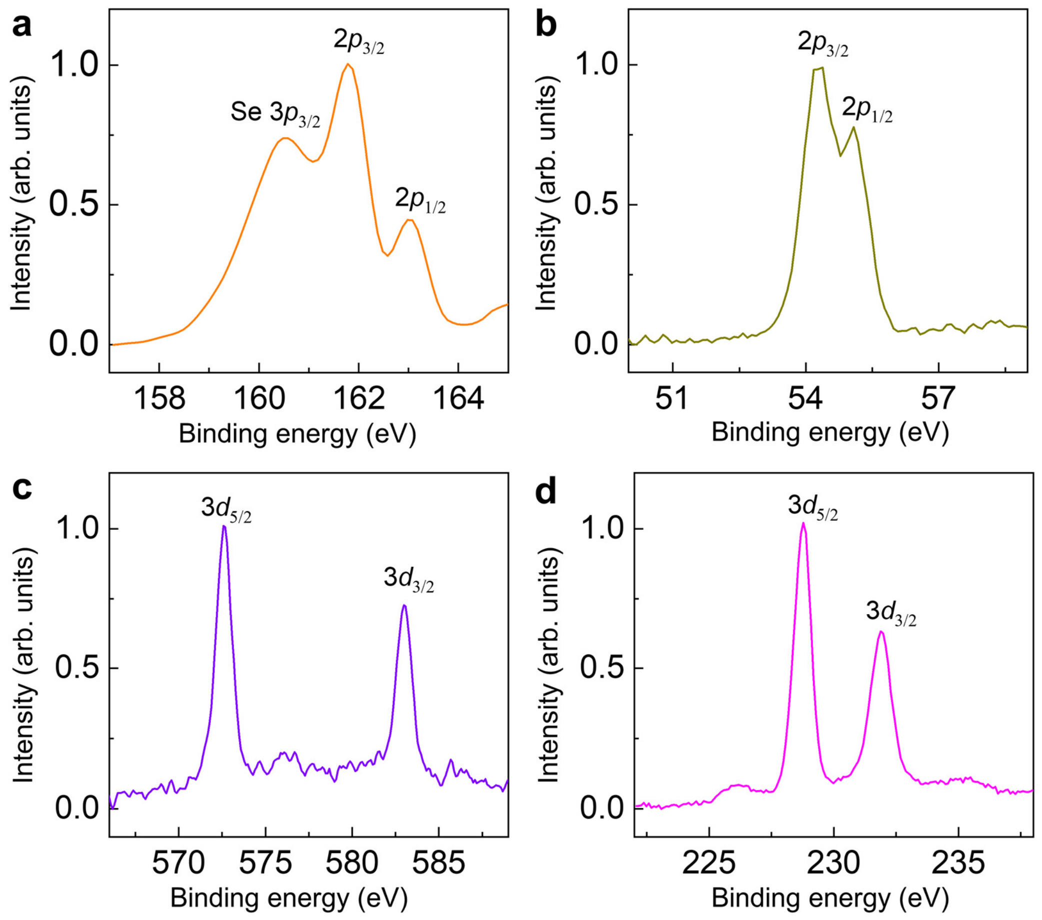


**Supplementary Fig. 11 | X-ray photoelectron spectroscopy (XPS) of quaternary 2D $MoS_{2(1-x-y)}Se_{2x}Te_{2y}$ alloy. a-d**, XPS spectra of as-grown $MoS_{2(1-x-y)}Se_{2x}Te_{2y}$ sample, showing binding energy states of Se 3*p* and S 2*p* (a), Se 2*p* (b), Te 3*d* (c), and Mo 3*d* (d), respectively.

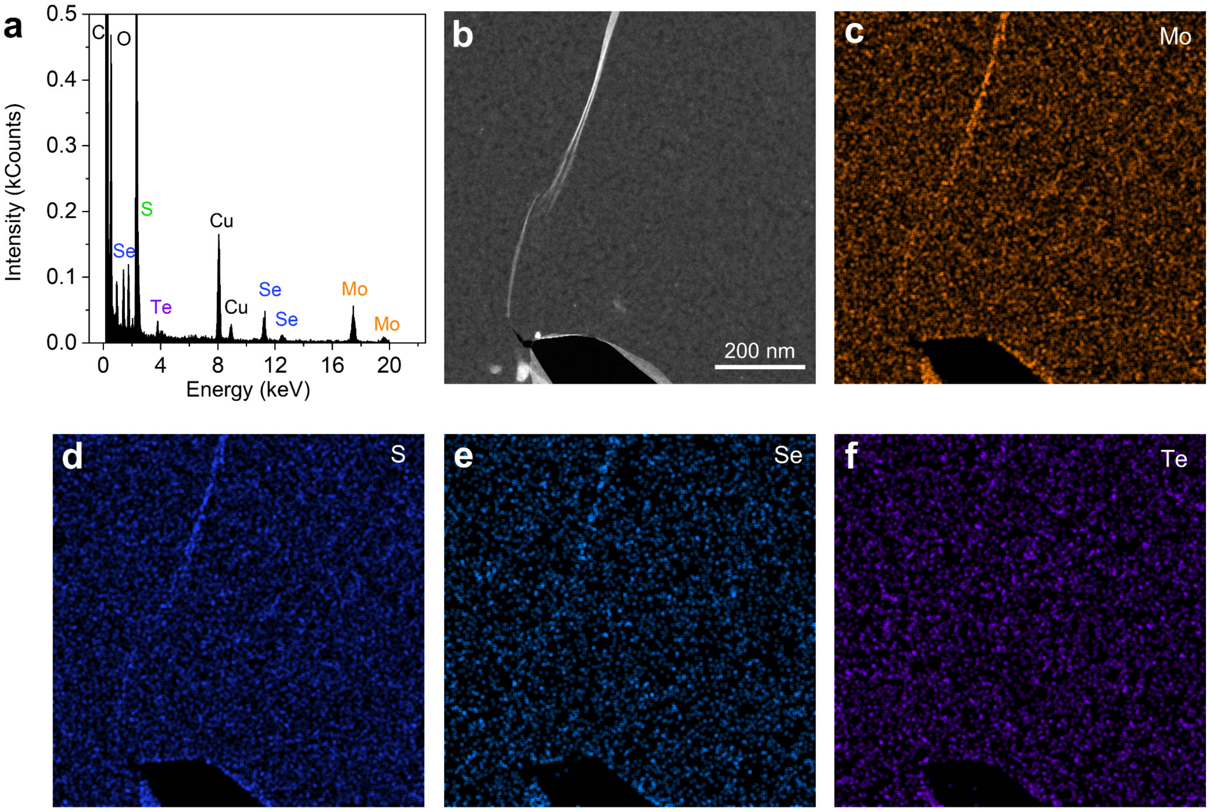


**Supplementary Fig. 12 | Energy Dispersive X-ray Spectroscopy (EDS) characterization of quaternary 2D $MoS_{2(1-x-y)}Se_{2x}Te_{2y}$ alloy. a**, EDS spectrum of $MoS_{2(1-x-y)}Se_{2x}Te_{2y}$ sample transferred onto TEM Cu grid. The peaks of S, Se, and Te were clearly observed, and their atomic concentrations were quantitatively extracted to be 84.0%, 12.7%, and 3.3%, respectively. **b**, The scanning transmission electron microscopy (STEM) image of the as-grown monolayer alloy film. **c-f**, EDS mapping of Mo (c), S (d), Se (e), and Te (f) corresponding to the area in (b), showing the uniform element distribution.

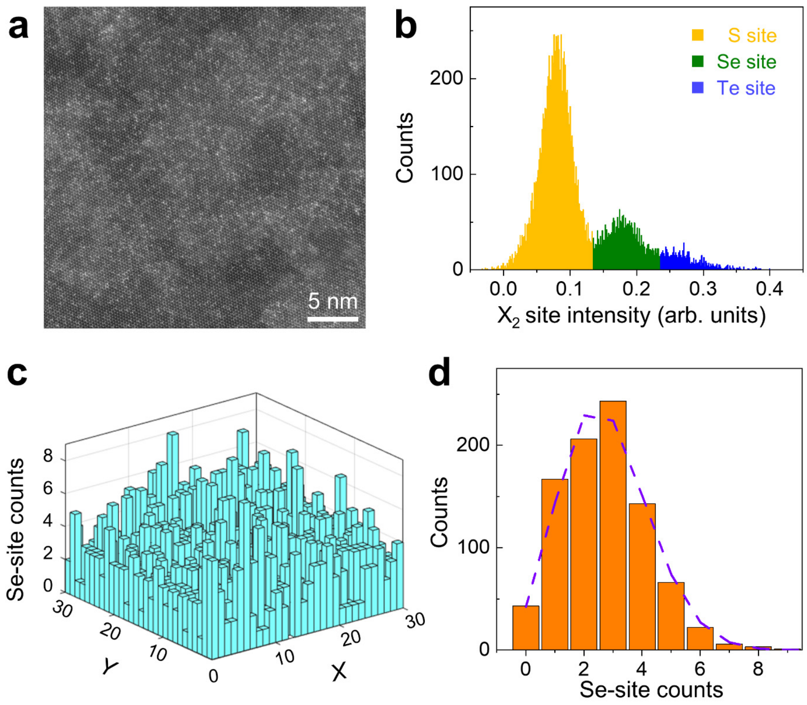


**Supplementary Fig. 13 | STEM analysis of quaternary 2D $MoS_{2(1\text{-}x\text{-}y)}Se_{2x}Te_{2y}$ alloy. a**, 32 × 32 nm$^2$ STEM image of $MoS_{2(1\text{-}x\text{-}y)}Se_{2x}Te_{2y}$ quaternary alloy. The Te-occupied sites in the image exhibit the brightest contrast and uniform distribution. **b**, Intensity histogram of the chalcogen atom ($X_2$) sites in (a). Due to the Z-contrast nature of STEM image, $X_2$ sites occupied by different atoms show distinct intensity ranges. The three peaks are assigned to be "S site", "Se site" and "Te site" regions as marked by yellow, green, and blue, respectively. The statistical result shows 9122 S sites, 2417 Se sites, and 771 Te sites from the total 12310 $X_2$ sites in (a). **c**, The Se-site distribution in the divided 30 × 30 parts of (a). **d**, The corresponding statistical histogram of Se-site counts in each part of (a). It shows a well binomial distribution feature (purple dotted line), revealing the random distribution of Se atoms.

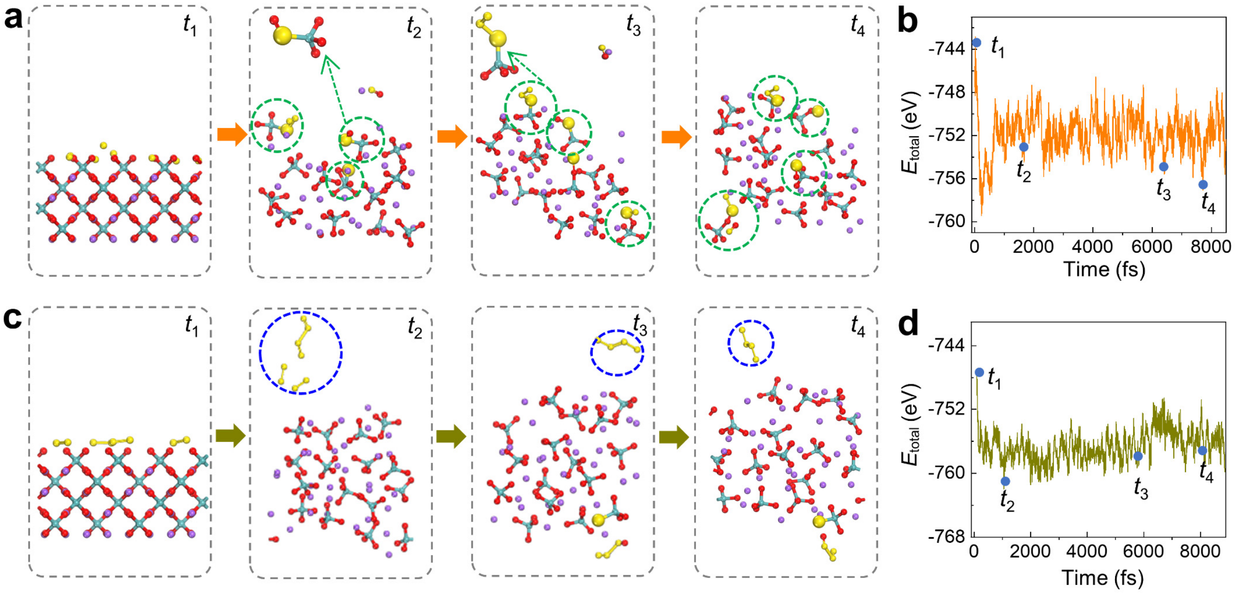


**Supplementary Fig. 14 | Snapshots taken during the molecular dynamic (MD) simulation of sulfidation of liquid $Na_2MoO_4$ and the corresponding energy profiles. a**,**b**, Snapshots taken during the MD simulation of sulfidation with eight sulphur monomers and the corresponding energy profiles at $t_1$ (0 ps), $t_2$ (1.69 ps), $t_3$ (6.27 ps) and $t_4$ (7.94 ps), respectively. It shows that S monomers can easily substitute the oxygen atoms of $Na_2MoO_4$. **c**,**d**, Snapshots taken during the MD simulation of sulfidation with four sulphur dimers and the corresponding energy profiles at $t_1$ (0 ps), $t_2$ (1.12 ps), $t_3$ (5.97 ps) and $t_4$ (8.00 ps), respectively. The structures in green circle presents the $MoS_xO_{4-x}$ cluster, the ones in blue circle indicate that S dimers disappears in the form of gas. The yellow, red, violet, and blue spheres represent sulphur, oxygen, sodium and molybdenum atoms, respectively.

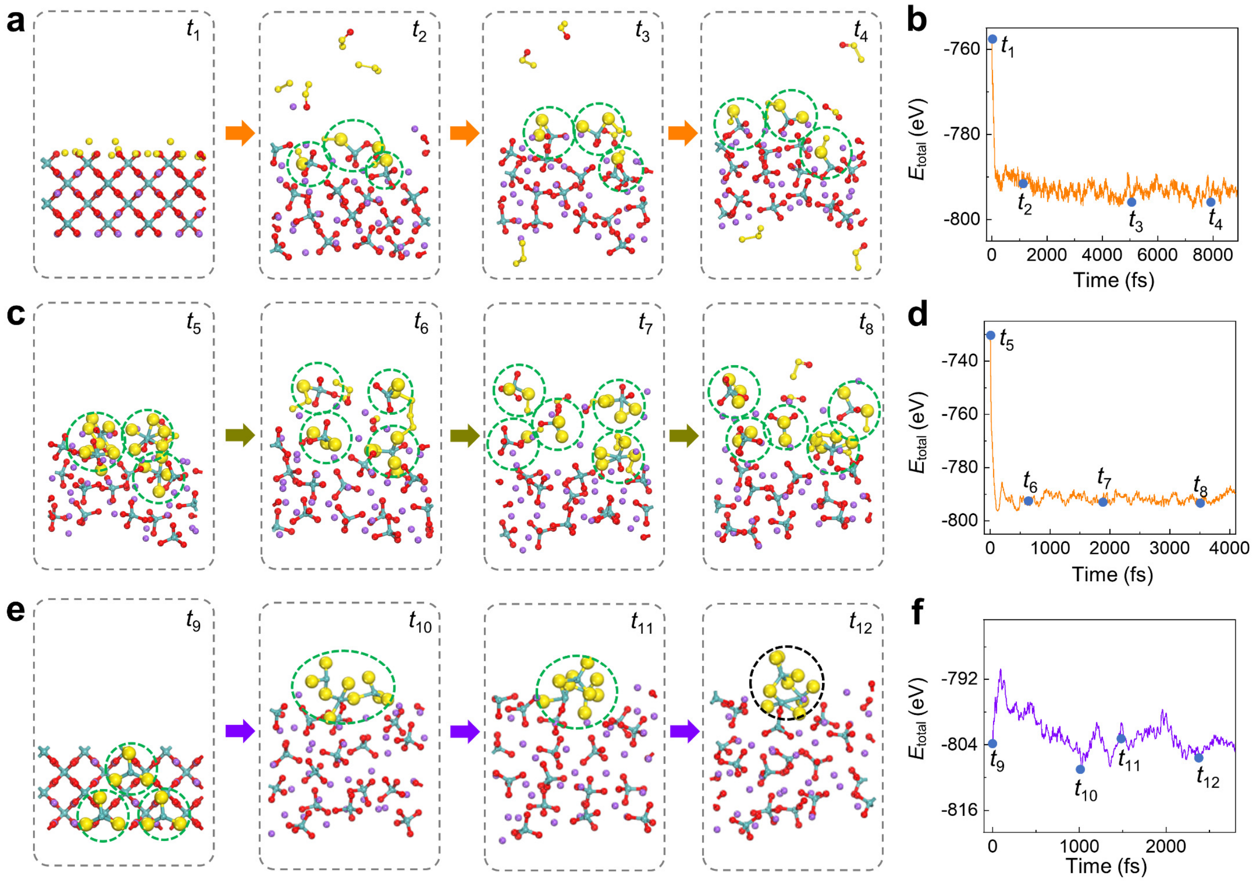


**Supplementary Fig. 15 | Snapshots taken during the MD simulation on the nucleation process with S monomers supply and their corresponding energy profiles. a**,**b**, Snapshots taken during the MD simulation when some sulphur monomers exist on the $Na_2MoO_4$ surface in the first stage and the corresponding energy profiles at $t_1$ (0 ps), $t_2$ (1.05 ps), $t_3$ (5.14 ps) and $t_4$ (8.00 ps), respectively. **c**,**d**, Snapshots taken during the MD simulation when $MoS_xO_{4-x}$ clusters (green circle) formed on the $Na_2MoO_4$ surface in the second stage and the corresponding energy profiles at $t_5$ (0 ps), $t_6$ (0.62 ps), $t_7$ (1.92 ps) and $t_8$ (3.50 ps), respectively. **e**,**f**, Snapshots taken during the MD simulation when $Mo_xS_y$ clusters (green circle) result in the nucleation of $MoS_2$ (black circle) in the finally stage and the corresponding energy profiles at $t_9$ (0 ps), $t_{10}$ (1.00 ps), $t_{11}$ (1.50 ps) and $t_{12}$ (2.48 ps), respectively. The yellow, red, violet, and blue spheres represent sulphur, oxygen, sodium, and molybdenum atoms, respectively.

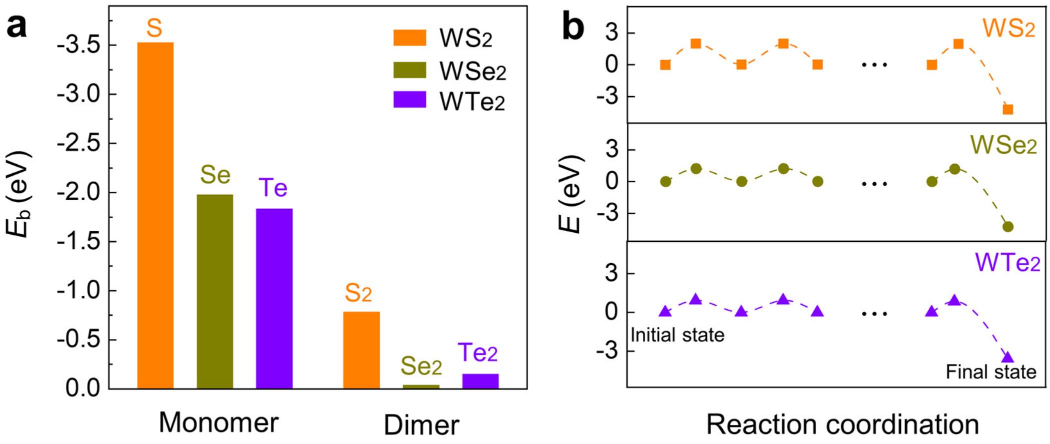


**Supplementary Fig. 16 | Proposed mechanism of chalcogen monomer modulated $WX_2$ (X = S, Se, Te) growth. a**, The binding energies of monomer and dimer on $MoX_2$ surface. **b**, The energy profiles of vacancy healing on $WX_2$ surface by using chalcogen monomers.

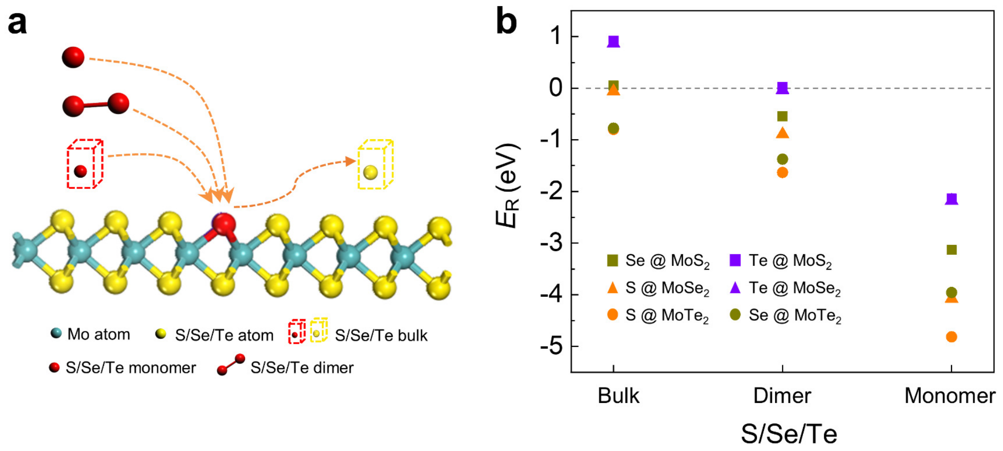


**Supplementary Fig. 17 | Calculated reaction energies of trinary TMD alloys. a**, The schematic diagram of substituting a chalcogen atom in a TMD by using chalcogen monomer, dimer and bulk as references. **b**, The reaction energies to form ternary TMD alloys. It can be clearly seen that the S, Se, and Te monomers can be easily doped into $MoX_2$ with reaction energies less than -2.0 eV, while doping of Te dimer or bulk into $MoS_2$/$MoSe_2$, S bulk into $MoSe_2$, Se bulk into $MoS_2$ are all difficult because of the near-zero or high reaction energies. These results further emphasize the advantage of S/Se/Te monomer supply to grow $MoS_{2(1-x-y)}Se_{2x}Te_{2y}$ alloy.

## Supplementary Table 1. Reported S vacancy density of as-grown monolayer TMDs

| Table S1 丨 Reported S vacancy density of as-grown monolayer TMDs | | | |
|---|---|---|---|
| **2D materials** | **Fabrication method** | **S vacancy density ($\times 10^{12}$ $cm^{-2}$)** | **Reference** |
| $MoS_2$ | ME | ~30 | 1 |
| $MoS_2$ | CVD | ~12±4 | 2 |
| $WS_2$ | CVD | 33±11 (interior)<br>92±45 (edge) | 3 |
| $WS_2$ | ME | 18.1 | 4 |
| $MoS_2$ | CVD | ~2 | This work |

Note: ME, mechanical exfoliation; CVD, chemical vapour deposition.

**Supplementary Note 1: Demonstrations on the supply rates of S monomer and Mo source.**

In the growth system, the S monomer is released from the ZnS surface and the Mo precursor is released from the $Na_2MoO_4$ at high temperature. Under the experimental conditions, the relationship between the concentrations of the S monomer or the Mo precursor and the temperature follows the Boltzmann distribution law. The ratio of S monomers to ZnS and Mo precursors to $Na_2MoO_4$ in the system are $N_{\mathrm{S}}/N_{\mathrm{ZnS}} = e^{(-\Delta E_1/K_B T)}$ and $N_{\mathrm{Mo}}/N_{\mathrm{Na_2MoO_4}} = e^{(-\Delta E_2/K_B T)}$, respectively, where $\Delta E_1$ and $\Delta E_2$ are the reaction potential energies and $K_B$ is the Boltzmann constant. $N_{ZnS}$ and $N_{\mathrm{Na_2MoO_4}}$ can be regarded as constant values because of sufficient supply. The release rates of S monomer and Mo precursor are positively related to their concentration in the system, thus can be expressed as

$$v_{\mathrm{S}} = k_1 e^{(-\Delta E_1/K_B T)}, \quad (1)$$

$$v_{\mathrm{Mo}} = k_2 e^{(-\Delta E_2/K_B T)}. \quad (2)$$

**Supplementary Note 2: Theoretical calculations on the landing probability of Mo precursor onto the substrate.**

To predict the nucleation density, we only consider the collision between the Mo source and the growth substrate. There are two types of contact between the Mo source and the substrate: collision and landing. The collision is regarded as completely elastic collision, and the direction is only changed when a collision occurs. While the landing processes describe the absorption of these molecules. Supposing that every molecule either scatters away from the area between the two substrates after multiple collisions, or gets absorbed by the growth substrate, the probability ($P_1$) of absorption during the landing process depends on the collision frequency *N*, that is,

$$P_1 = \alpha \cdot \frac{1-(1-\alpha)^{2N}}{1-(1-\alpha)^2}. \tag{3}$$

Considering the velocity directions of all Mo precursors, the collision frequency (*N*) of the Mo precursor in direction $\theta$ at a certain distance of *d* can be obtained by dividing the average of the distances moved by all Mo precursors in the Z-direction before leaving the area by *2d*, that is,

$$N = \frac{L}{4d tan\theta}, \tag{4}$$

where *L* is the length of the substrate and $\alpha$ is the landing probability in one collision. For these Mo precursors in the semi solid angle, the landing probability (*P*) can be calculated by an integral,

$$P = \int_0^{\frac{\pi}{2}} \alpha \cdot \frac{1-(1-\alpha)^{2 \cdot \frac{L}{4d\tan\theta}}}{1-(1-\alpha)^2} \sin\theta d\theta. \tag{5}$$

Therefore, the relationship between the landing probability (*P*) and *d* for Mo precursor can be described as shown in Fig. 1d.

**Supplementary Note 3: Calculations of the formation energies of different transition metal dichalcogenides.**

To understand the relative stability of these TMDs ($MX_2$, M = Mo, W; X = S, Se, Te), the formation energies of these TMDs were calculated by using

$$E_{\mathrm{f}} = E_{\mathrm{MX_2}} - \mu_{\mathrm{M}} - 2\mu_{\mathrm{X}}, \quad (6)$$

where $E_{\mathrm{MX_2}}$ is the energy of the $MX_2$, $\mu_{\mathrm{M}}$ and $\mu_{\mathrm{X}}$ are the energies of M in the form of bulk and X atoms using monomer or bulk as references, respectively. These results on formation energies (Fig. 2f) clearly show that $MS_2$ and $MSe_2$ are highly stable, while $MTe_2$ might hardly to be synthesized due to their high formation energies, which agrees well with the most experimental results of TMD synthesis[5, 6].

**Supplementary Note 4: Statistical Se and Te atom distribution in the quaternary $MoS_{2(1-x-y)}Se_{2x}Te_{2y}$ alloy.**

To evaluate the distribution of Se and Te atoms in our quaternary alloy, statistical analysis was conducted in a 32 × 32 $nm^2$ STEM image (Supplementary Fig. 13a). The atoms in the image were sorted into metal atom (M) sites and chalcogen atom ($X_2$) sites. The intensity histogram of all $X_2$ sites shows three peaks, which are assigned to be "S site", "Se site" and "Te site" regions, respectively, according to the Z-contrast nature of STEM image (Supplementary Fig. 13b). Note that every $X_2$ site in STEM image is actually a projection of two overlapped X atoms along the electron beam direction, "S site" denotes both X atoms are S, "Se site" means two possible configurations: Se-S or Se-Se, while "Te site" stands for Te-S, Te-Se, or Te-Te. According to the overall statistical result, *P*(S), the probability for an $X_2$ site to be "S site", is 9122/(9122+2417+771) = 0.741. Similarly, the probability of "Se site" and "Te site" denoting as *P*(Se) and *P*(Te), are 0.196 and 0.063, respectively.

Assume that Se and Te are randomly distributed in the $X_2$ sites, the overall probability of *P*(Se) and *P*(Te) will also be valid to every $X_2$ site. Thus, the probability of Se distribution could be calculated by the binomial distribution

$$f(k; N, P(\mathrm{Se})) = \frac{N!}{k!\,(N-k)!} P(\mathrm{Se})^k (1 - P(\mathrm{Se}))^{N-k}, \tag{7}$$

where *N* is the total $X_2$ site number of a region and *k* is the "Se site" number found in it. The same analysis can be applied to the Te case. To verify this random-distribution assumption, the entire image was cut into identical 30 × 30 parts, each of which comprised about 14 $X_2$ sites. The statistical histograms of "Te site" and "Se site" for these 900 parts are shown in Fig. 3i and Supplementary Fig. 13d, which match well with the purple dotted lines calculated by the binomial distribution, suggesting the random distribution of Te and Se atoms in our quaternary alloy.

In addition, there is a relationship between the probability *P* and the atom concentration *c*,

$$P(\mathrm{S}) = c(\mathrm{S})^2 \tag{8}$$

$$P(\mathrm{Se}) = 2c(\mathrm{Se})c(\mathrm{S}) + c(\mathrm{Se})^2 \tag{9}$$

$$c(\mathrm{S}) + c(\mathrm{Se}) + c(\mathrm{Te}) = 1. \tag{10}$$

From the probability data and these equations, the concentrations of S, Se and Te were extracted to be 86.1%, 10.7%, 3.2%, respectively, which are very close to the concentrations extracted from the preceding EDS spectrum (84.0%, 12.7%, and 3.3%, respectively, as mentioned in Supplementary Fig. 12) and confirm our statistical conclusion.

**Supplementary Note 5: Theoretical calculations on initiated nucleation of $MoS_2$ by sulphur monomer or dimer supply.**

Based on the MD simulations, the sulfidation processes of $Na_2MoO_4$ are analysed at atomic-level. Here we consider the sulphur source in the form of S monomers and $S_2$ dimers respectively. As shown in Supplementary Fig. 14, eight S monomers and four $S_2$ dimers were deposited on the $NaMoO_4$ surface during the MD trajectories. The MD simulations clearly showed that S monomers are more active and can easily substitute the oxygen atoms of $Na_2MoO_4$, resulting in SO or $SO_2$ molecules in the gas phase and Mo-S bond formation in the liquid $Na_2MoS_xO_{4-x}$ (green circle). This proves that S monomers released from the ZnS surface are more reactive in the growth of TMDs. Adding more S monomers to the $Na_2MoO_4$ surface (Supplementary Fig. 15a,b), the aggregation of Mo and S atoms during the MD simulation is clearly seen and the clusters of $MoS_3O_x$ are formed on the surface (Supplementary Fig. 15c,d). To demonstrate the nucleation of $MoS_2$, we added a few $MoS_3$ molecules on the substrate and large $Mo_xS_y$ clusters (green circle) were formed during the MD simulation (Supplementary Fig. 15e,f). In summary, we have demonstrated the mechanism of TMD growth by the density-functional-theory-based MD simulations. Firstly, the active S monomers replace O atoms in $Na_2MoO_4$. Then, the high concentration of S substitutes in the $Na_2MoO_4$ will lead to the formation of $Mo_xS_y$ clusters on the surface of the substrate. Finally, the aggregation of $Mo_xS_y$ clusters results in the nucleation of $MoS_2$ on the $Na_2MoO_4$ surface.

**Supplementary Note 6: Reaction energies of ternary TMD alloys obtained by X (X = S, Se, Te) doped $MoX_2$.**

In order to confirm the possibility of forming $MoS_{2(1-x-y)}Se_{2x}Te_{2y}$ alloy, we calculated the reaction energy ($E_R$) of X (X = S, Se, Te) doped $MoX_2$ (X = S, Se, Te). Here, we take ternary alloy $MoSe_xS_{2-x}$ as an example. Its reaction energy can be calculated by using

$$E_{\mathrm{R}} = E_{\mathrm{MoSe}_x\mathrm{S}_{2-x}} - E_{\mathrm{MoS}_2} + x\mu_S - x\mu_{\mathrm{Se}}, \quad (11)$$

where $E_{\mathrm{MoSe}_x\mathrm{S}_{2-x}}$ and $E_{\mathrm{MoS}_2}$ are the energies of ternary alloy $MoSe_xS_{2-x}$ and perfect $MoS_2$, respectively. $\mu_S$ and $\mu_{Se}$ are the energies of S and Se atoms which are in the form of bulk, dimer and monomer, respectively. The obtained different formation energies are shown in Supplementary Fig. 17. It is found that the monomers of S, Se and Te monomers can be easily doped into $MoX_2$ with reaction energies less than -2.0 eV. While the $Te_2$ could hardly be used to dope $MoS_2$ or $MoSe_2$ because of the near zero reaction energy. If bulk dopants are employed, only S/Se can easily dope the $MoTe_2$. This result further confirms the advantage of using S/Se/Te monomer to grow $MoS_{2(1-x-y)}Se_{2x}Te_{2y}$ alloys.

## References


1. Qiu, H. et al. Hopping transport through defect-induced localized states in molybdenum disulphide. *Nat. Commun.* **4**, 2642 (2013).

2. Hong, J. et al. Exploring atomic defects in molybdenum disulphide monolayers. *Nat. Commun.* **6**, 6293 (2015).

3. Carozo, V. et al. Optical identification of sulfur vacancies: Bound excitons at the edges of monolayer tungsten disulfide. *Sci. Adv.* **3**, e1602813 (2017).

4. Jiang, J. et al. A facile and effective method for patching sulfur vacancies of $WS_2$ via nitrogen plasma treatment. *Small* **15**, 1901791 (2019).

5. Empante, T. A. et al. Chemical vapor deposition growth of few layer $MoTe_2$ in the 2H, 1T', and 1T phases: Tunable properties of $MoTe_2$ Films. *ACS Nano* **11**, 900-905 (2017).

6. Li, J. et al. Centimeter-scale, large-area, few-layer 1T'-$WTe_2$ films by chemical vapor deposition and its long-term stability in ambient condition. *J. Phys. Chem. C* **122**, 7005-7012 (2018).
